# Opportunities and Challenges in Developing a *Cryptosporidium* Controlled Human Infection Model for Testing Antiparasitic Agents

**DOI:** 10.1021/acsinfecdis.1c00057

**Published:** 2021-04-06

**Authors:** Rajiv S. Jumani, Johanne Blais, Hanns-Christian Tillmann, Florencia Segal, Dean Wetty, Christian Ostermeier, Natko Nuber, Jay Lakshman, Natasha Aziz, Richa Chandra, Wilbur H. Chen, Cynthia L. Chappell, Thierry T. Diagana, Ujjini H. Manjunatha

**Affiliations:** + Novartis Institute for Tropical Diseases, Novartis Institutes for BioMedical Research, Inc., Emeryville, California 94608-2916, United States; △ Novartis Institutes for BioMedical Research, Inc., Translational Medicine, 4056 Basel, Switzerland; § Novartis Institutes for BioMedical Research, Inc., Cambridge, Massachusetts 02139-4133, United States; ∥ Novartis Pharma AG, 4002 Basel, Switzerland; ⊥ Novartis Pharmaceuticals Corporation, East Hanover, New Jersey 07936, United States; # Center for Vaccine Development and Global Health, University of Maryland School of Medicine, Baltimore, Maryland 21201, United States; □ The University of Texas School of Public Health Houston, Texas 77225-0186, United States

**Keywords:** diarrhea, cryptosporidiosis, human-challenge model, drug discovery, *Cryptosporidium*, pediatric development, antiparasitic agent, CHIM

## Abstract

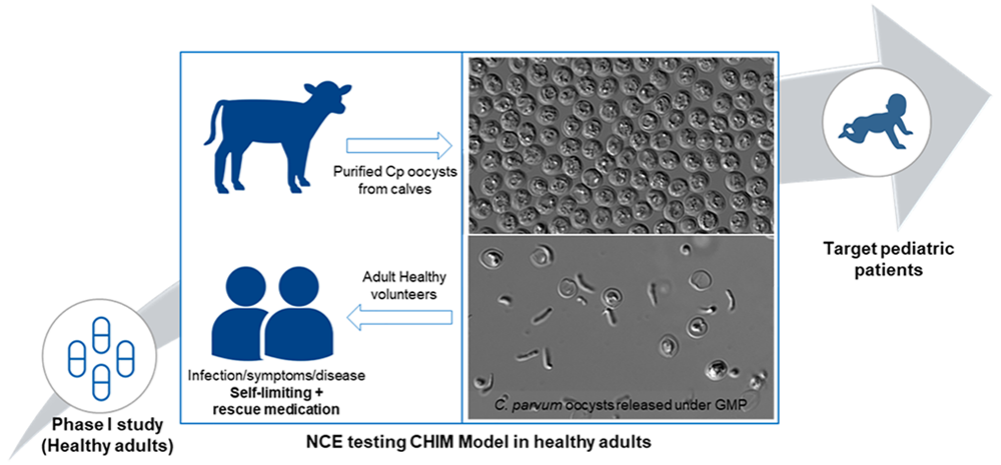

Cryptosporidiosis is a leading cause of moderate-to-severe diarrhea in low- and middle-income countries, responsible for high mortality in children younger than two years of age, and it is also strongly associated with childhood malnutrition and growth stunting. There is no vaccine for cryptosporidiosis and existing therapeutic options are suboptimal to prevent morbidity and mortality in young children. Recently, novel therapeutic agents have been discovered through high-throughput phenotypic and target-based screening strategies, repurposing malaria hits, etc., and these agents have a promising preclinical in vitro and in vivo anti-*Cryptosporidium* efficacy. One key step in bringing safe and effective new therapies to young vulnerable children is the establishment of some prospect of direct benefit before initiating pediatric clinical studies. A *Cryptosporidium* controlled human infection model (CHIM) in healthy adult volunteers can be a robust clinical proof of concept model for evaluating novel therapeutics. CHIM could potentially accelerate the development path to pediatric studies by establishing the safety of a proposed pediatric dosing regimen and documenting preliminary efficacy in adults. We present, here, perspectives regarding the opportunities and perceived challenges with the *Cryptosporidium* human challenge model.

## Cryptosporidiosis Medical Need

*Cryptosporidium* spp. are protozoan parasites responsible for acute enteritis with diarrhea as the primary clinical symptom. Cryptosporidiosis in humans is caused primarily by two species, *Cryptosporidium parvum* and *Cryptosporidium hominis*.^1^ Transmission typically occurs when feces containing *Cryptosporidium* oocysts from infected animals or humans contaminate food or water supplies, thereby infecting humans predominantly via the fecal oral route. Once ingested, the oocysts reach the small intestine, where motile, infectious sporozoites are released and infect intestinal epithelial cells. The organisms then goes through multiple cycles of asexual replication, followed by sexual reproduction, ultimately resulting in excretion of numerous mature oocysts in the feces.^2^ During a single infection period individuals may shed up to 10^8^–10^9^ oocysts.^3^

Cryptosporidiosis is a self-limiting infection in immunocompetent adults and can be successfully managed with supportive care and treatment. In the vulnerable patients population (young children and immunocompromised adults), *Cryptosporidium* infection is associated with prolonged (7–14 days) or persistent (>14 days) diarrhea.^4^ Indeed, *Cryptosporidium* spp. are a leading cause of pediatric diarrhea in low- and middle-income countries (LMICs) and represents one of the leading causes of diarrheal deaths in young children aged 0–24 months.^5−8^ Cryptosporidiosis is estimated to be responsible for 48 000–202 000 deaths annually in children younger than two years of age in South Asia and Sub-Saharan Africa and ∼7.6 million diarrhea cases annually are attributable to *Cryptosporidium* infection in these regions.^5,9^ In addition, evidence suggests that repeated *Cryptosporidium* infections in children are associated with long-term effects and debilitating growth-stunting.^10,11^

Nitazoxanide (Alinia) is the only drug approved by the U.S. Food and Drug Administration for the treatment of cryptosporidiosis in children aged 1 year of age and older and immunocompetent adults.^12,13^ It is a safe oral antiparasitic agent and significantly improves clinical response and reduces the duration of diarrhea and oocyst shedding in immunocompetent adults with cryptosporidiosis.^14,15^ As a parasitistatic agent,^16^ efficacy of nitazoxanide is largely dependent on host-immunity and is not effective for treating cryptosporidiosis in immunocompromised patients.^17,18^ In a study that enrolled HIV-negative, malnourished children, nitazoxanide treatment resulted in resolution of diarrhea in only 56% of children (23% in placebo group) and only 52% demonstrated oocyst clearance (14% in placebo group).^19^ The limited efficacy of nitazoxanide in malnourished children may be attributed to immunological alterations or intestinal dysbiosis associated with malnutrition in these children.^20,21^ Overall, there is a pressing, highly unmet therapeutic need to address enteric cryptosporidiosis in three major target patient populations: young children aged 0–24 months in LMICs, malnourished children under age five, and immunosuppressed individuals of any age.^22^

## Anti-*Cryptosporidium* Drug Discovery and Development Efforts

Despite the substantial global disease burden and a clear need for effective antiparasitic treatments, cryptosporidiosis remains an under-appreciated global health concern. Earlier efforts to repurpose approved drugs, such as paromomycin, rifamycin, spiramycin, azithromycin, letrazuril, HIV protease inhibitors, or clofazimine, for the treatment of cryptosporidiosis in HIV-AIDS patients have been unsuccessful.^1,23^ Recently, significant progress has been made in identifying and optimizing diverse new chemical entities (NCEs) with promising in vitro activity and in vivo efficacy as defined in the proposed target product profile for cryptosporidiosis treatment.^24,25^ Some of the promising NCEs include *Cryptosporidium* calcium-dependent protein kinase 1 (CpCDPK1) inhibitors,^26^ phosphatidylinositol-4-OH kinase (PI(4)K) inhibitors,^27^ piperazine-based lead compound MMV665917,^16^ lysyl-tRNA synthetase (KRS) inhibitors,^28^ oxaboroles that are a cleavage and polyadenylation specificity factor3 inhibitors,^29,30^ bicyclic azetidines that are phenylalanyl-tRNA synthetase inhibitors,^31^ methionyl-tRNA synthetase inhibitors,^32^ a choline-based phospholipid VB-201,^33^ and multiple novel cell-active hits.^34^ Most of these NCEs have demonstrated antiparasitic activity against both *C*. *parvum* and *C*. *hominis*. Further, unlike nitazoxanide, many of these anti-*Cryptosporidium* NCEs are effective in reducing the fecal oocyst burden in immunocompromised mouse models. This rich and diverse pipeline of drug candidates is very encouraging and could also enable drug combinations to address the potential for drug resistance.^32^

To address the unmet medical need in the highly vulnerable young pediatric cryptosporidiosis patient population, the most critical aspects are an exceptional safety profile and robust efficacy demonstrated by rapid resolution of diarrhea to minimize the risk of dehydration. A few candidate molecules such as the CDPK inhibitor BKI-1369,^26^ PI(4)K inhibitor KDU731,^27^ MMV6659917,^16^ and 6-carboxamide benzoxaborole AN7973^29^ have demonstrated promising activity in resolving diarrheal symptoms in neonatal calves, a preclinical model of cryptosporidiosis diarrhea which closely resemble pediatric infection and illness. Overall, in the past few years, substantial progress has been made in identifying diverse NCEs, and it is anticipated that some may soon start clinical development.

## Challenges in Developing a Novel Antiparasitic Agent to Treat Pediatric Cryptosporidiosis

Cryptosporidiosis disproportionately affects young children, and the highest unmet medical need is in the malnourished who are at the greatest risk for severe disease and mortality.^22^ Drug development is expensive, takes considerable time, has a high attrition rate and the pediatric population in LMICs presents additional challenges. Four study populations to establish proof of concept (PoC) of new anti-*Cryptosporidium* compounds are possible: (i) adult immunocompromised patients in LMICs; (ii) adult patients during a sporadic outbreaks; (iii) malnourished pediatric patients in endemic regions; and (iv) a *Cryptosporidium* controlled human infection model (CHIM) in healthy adult volunteers. Typical drug development and regulatory pathways involve demonstrating the prospect of direct benefit in adult populations before initiating pediatric studies.^35^

*Cryptosporidium* infection is a common cause of chronic diarrhea in HIV/AIDS patients in LMICs.^36^ Currently, the best treatment is reconstitution of the immune response via antiretroviral therapy. HIV/AIDS patients are the only naturally occurring adult population of adequate size to facilitate early stage drug efficacy studies for cryptosporidiosis in LMICs. However, a recent controlled clinical trial to assess the safety and efficacy of clofazimine for the treatment of cryptosporidiosis in this population highlighted the significant challenges with this approach.^23^ In addition to the operational complexity of conducting early phase clinical trials in resource-poor settings,^37^ safety and efficacy evaluation in the HIV/AIDS cryptosporidiosis patient population is highly confounded by the severe immunocompromised state, presence of multiple diarrheal pathogens, other opportunistic coinfections, concurrent medications, failure of antiretroviral therapy, and high mortality.^38^ As a potential alternative to investigating NCEs in HIV/AIDS cryptosporidiosis coinfected patients, a *Cryptosporidium* CHIM in adult healthy volunteers is considered herein as it offers a scientifically robust path to PoC for novel antiparasitic agents. In CHIM, the infectious pathogen is administered to healthy adult volunteers with the intent to deliberately induce infection and clinical symptoms in a controlled setting. Novel therapies can then be evaluated in a randomized blinded treated cohort and compared to the clinical symptoms and disease duration in the untreated (placebo-treated) cohort. Across different infectious diseases, CHIM studies have played a very important role for the understanding of disease mechanisms and also for establishing PoC for drug and vaccine development.^39−41^ A sporadic outbreak of cryptosporidiosis is not a feasible option for structured drug development due to its anticipated protracted time period. Table 1 summarizes the pros and cons of the other three PoC human efficacy studies, namely, *Cryptosporidium* CHIM, HIV-positive adult cryptosporidiosis patients and pediatric cryptosporidiosis patient populations for testing novel anti-*Cryptosporidium* NCEs.

**Table 1:** Pros and Cons of Potential First in Human Proof of Concept Efficacy Studies for Testing a Novel Anti-*Cryptosporidium* NCEs

	pros	cons
*Cryptosporidium* controlled human infection model (CHIM) in healthy adults	· prospect of benefit in healthy adults with *Cryptosporidium* induced diarrhea	· *C*. *parvum* model utilized for technical reasons, although *C*. *hominis* more common human pathogen
	· informs dose selection for studies in pediatric patients	· needs to be established and validated
	· clinical syndrome, parasitological and clinical end points under monoinfection condition	· limited viability period of GMP oocysts
	· conducted in healthy volunteers, mitigates safety confounders	· monoinfection state may not be clinically relevant to target pediatric patient population
	· phase 1 settings: faster recruitment and smaller sample size	· unknown translatability of efficacy to target population
Adult HIV-positive cryptosporidiosis patients	· natural infection in potential secondary target population	· confounded safety and efficacy due to advanced immunocompromised state
	· PK in context of high GI motility	· presence of other pathogens/coinfections and/or concurrent medications
		· high mortality
		· operational complexity in the resource poor settings
Pediatric cryptosporidiosis patient population	· assessment of safety and efficacy in the target population	· prospect of clinical benefit will not have been previously established
	· natural course of infection	· high-risk and vulnerable patient population
	· with relevant clinical strains	· uncertainty in predicted efficacious dose in the context of high GI motility
		· risk investment in juvenile toxicity study prior to phase I to avoid program delays
		· operational complexity in the resource poor settings

## Establishing a *Cryptosporidium* CHIM to Enable Drug Discovery

More than 15 species of *Cryptosporidium* are known to cause human infection with two predominant clinical species, *C*. *hominis* (∼80%) and *C*. *parvum* (∼10%).^9^ The safety and feasibility of controlled human *Cryptosporidium* challenge studies are well documented in the literature, using *C*. *parvum*,^42−48^*C*. *hominis*,^49^*C*. *meleagridis*,^50^ and *C*. *muris*.^51^ These challenge studies focused on: identifying the minimum human infectious dose for *C*. *parvum* and *C*. *hominis*; comparing the clinical symptoms caused by different clinical isolates; understanding the impact of prior *Cryptosporidium* infection on rechallenge; assessing fecal inflammatory markers; and exploring mechanisms of pathogenesis (Table 2). To date, more than 200 healthy adult volunteers have been challenged with *Cryptosporidium* oocysts, of which ∼175 were infected with various isolates of *C*. *parvum*. Among them, *C*. *parvum* Iowa isolate from the University of Arizona was used in 5 of the 7 published studies.^43,44,46−48^ The *C*. *parvum* Iowa isolates used were not from a single oocyst stock, but were continuously propagated in calves, which could lead to mutations and/or genetic drift in oocysts over time. In these CHIM studies, a high percentage of infections with *Cryptosporidium* could be elicited and many of the infected individuals developed clinical symptoms after challenge (summarized in Table 2). In addition, no safety concerns (other than clinical symptoms of acute cryptosporidiosis) have been observed in any of the CHIM studies with doses up to 10^6^ oocysts.

**Table 2:** Summary of the Published *Cryptosporidium* CHIM Studies

species isolate	study reference	dose	*N*	infection^a^ (%)	illness^a^ (%)	notes
*C*. *parvum* Iowa	DuPont, NEJM 1995	3 × 10^1^–10^6^	29	20–100	0–38	· first *Cryptosporidium* human challenge study; ID_50_ established 132 oocysts
						· oocyst purified from neonatal calves
						· self-limited infection and illness observed; at dose ≥1000 oocysts: 100% infection, 71% enteric symptoms and 29% diarrheal illness observed
*C*. *parvum* Iowa	Okhuysen, Inf Imm 1998	5 × 10^2^	19	84	58	· rechallenge (extension of DuPont 1995) study in healthy adults
						· fewer subjects shed oocysts after the second exposure (16%) than after the first exposure (63%)
						· lower “intensity of diarrhea” with rechallenge
*C*. *parvum* Iowa	Chappell, AJTMH 1999	5 × 10^2^–5 × 10^4^	17	41	59	· infectivity in pre-existing anti-*C*. *parvum* serum IgG
						· ID_50_ is 1880 oocysts, 20× higher than in seronegative volunteers
						· prior exposure provides protection from infection and illness at low oocyst doses
*C*. *parvum* Iowa	Okhuysen, JID 1999	3 × 10^1^–10^5^	29	40–100	52	· virulence of 3 *C*. *parvum* isolates compared, Iowa and UCP, originally isolated from calves and passaged in calves; whereas TAMU isolated from a student who got infected from foal, also passaged in calves
*C*. *parvum* UCP		5 × 10^2^–10^4^	17	10–100	59	· ID_50_ for Iowa, UCP, and TAMU established as 87, 1042, and 9 oocysts, respectively, based on presumed infection
*C*. *parvum* TAMU		10^1^–5 × 10^2^	14	0–100	86	· TAMU isolate induced higher diarrhea rate for a longer duration than Iowa or UCP isolates
*C*. *parvum* Iowa/TAMU	Alcantara, AJTMH 2003	10^2^–10^3^	15	50–67	33–83	· importance of intestinal inflammation in *C*. *parvum* challenge versus pediatric patients as measured by fecal IL8, lactoferrin, and TNFα
						· significantly more inflammation in pediatric *Cryptosporidium* patients than adult volunteers
*C*. *parvum* UCP	Okhuysen, CID 1998	5 × 10^3^–1 × 10^4^	20	44–100	56–75	· prophylactic effect of bovine hyperimmune anti-*Cryptosporidium* colostrum
						hyperimmune colostrum not protective against infection
*C*. *parvum* Moredun	Okhuysen, JID 2002	10^2^–3 × 10^3^	16	33–75	60–75	· oocysts originally isolated from red deer, passaged in sheep and later in calves
						· ID_50_ 300 oocysts; diarrheal illness was frequently associated with oocyst excretion
**Healthy Volunteers Challenged with *C. parvum*: *N* = 176**						
						
*C*. *hominis* TU502	Chappell, AJTMH 2006	10^1^–5 × 10^2^	21	20–80	40–75	· first *C*. *hominis* human challenge study, ID_50_ established 10–83 oocysts
						· oocyst purified from gnotobiotic piglets
						· infection and illness similar to *C*. *parvum* challenge studies
*C*. *meleagridis*	Chappell, AJTMH 2011	10^5^	5	100	80	· first *C*. *meleagridis* human high-dose challenge study
						· oocyst purified from gnotobiotic piglets
						· caused self-limited infection and mild diarrheal illness
*C*. *muris*	Chappell, AJTMH 2015	10^5^	6	100	33	· first *C. muris* human high-dose challenge study
						· oocyst purified from Nu/Nu mouse
						· caused persistent infection and self-limited diarrheal illness
						· persistent shedders were treated with nitazoxanide, and the infection was resolved

a Note: In most studies, “illness” was defined as the passage of 3 unformed stools in 8 h or >3 unformed stools in 24 h accompanied by the presence of one or more enteric symptoms, including fever, nausea, vomiting, abdominal pain or cramps, and gas-related intestinal symptoms. “Infection” was defined as the excretion of oocysts in stool by a direct immunofluorescence assay (DFA) after a flow-through period of 36 h postchallenge.

**Figure 1:**
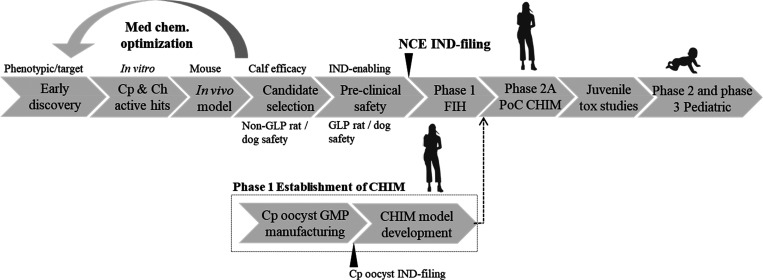
Pediatric cryptosporidiosis drug discovery and development, a proposed path to registration. Development of *C*. *parvum* oocyst CHIM is shown above. Cp, *C*. *parvum* and Ch, *C*. *hominis*.

**Figure 2:**
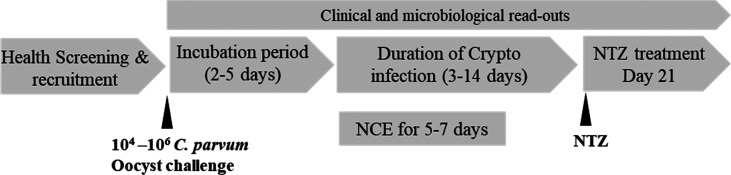
Proposed controlled human *C*. *parvum* high-dose infection model for testing NCE with anticipated incubation period and duration of infection. NTZ, nitazoxanide.

We propose establishing a *C*. *parvum* Iowa isolate high-dose oocyst human challenge model to enable future assessment of NCEs for the treatment of cryptosporidiosis. The proposed path for pediatric cryptosporidiosis drug discovery and development incorporates CHIM for establishing PoC for efficacy in adults (Figure 1). The synopsis of a CHIM study design is further outlined in Figure 2. Following *Cryptosporidium* challenge, healthy, immunocompetent individuals may experience profuse, watery, nonbloody diarrhea after an incubation period of 3–12 days. Without any treatment, symptoms are expected to resolve within 2–3 weeks or less (mean duration of 12.7 days) but could persist for up to a month. Thus, to minimize the risk of long-term asymptomatic shedding and/or recurrence, all subjects with elicited infection will receive nitazoxanide treatment, the standard of care, at the end of the 21-day study. Of note, as a precaution any subjects who remain asymptomatic postchallenge will also be treated to prevent any potential secondary transmission. Once CHIM is established, it could potentially be used for a NCE development after phase I studies. The following section highlights the opportunities and challenges with *Cryptosporidium* CHIM in healthy adult volunteers, a model designed for establishing efficacy with NCEs.

## Opportunities and Challenges in *Cryptosporidium* CHIM

Some of the major advantages of *Cryptosporidium* CHIM are as follows:

- It enables the assessment of the prospect of benefit in adults before NCE is advanced into efficacy studies in the vulnerable pediatric patient population in the LMICs.
- Subjects in a cryptosporidiosis CHIM will have a typical noninflammatory diarrhea because of a single pathogen, allowing for unconfounded interrogation of the effect of an investigational drug on clinical and parasitological end points.
- It allows for careful and extensive analysis of the pharmacokinetic–pharmacodynamic (PK–PD) relationship of an investigational drug in the presence of diarrhea, providing valuable data that informs dose selection for future pediatric clinical trial designs.
- *Cryptosporidium* challenges induce nonlife threatening, self-limiting infections in healthy adults with the option to use nitazoxanide as the rescue medication.
- Finally, CHIM PoC efficacy studies can help prioritize NCEs for juvenile toxicity studies designed to understand potential adverse effects on postnatal growth and development, thereby hastening the pediatric clinical development.

Overall, *Cryptosporidium* CHIM may enable scientifically rigorous and rapid clinical development path for novel drug candidates to treat cryptosporidiosis in young children. However, there are some challenges in establishing and utilizing a *Cryptosporidium* CHIM for drug development. Some of these challenges and mitigation strategies are described below.

### Challenge Organisms Are Regulated As Biological Products and Drugs in the US

According to a 2013 guidance from the US Food and Drug Administration (FDA), an Investigational New Drug Application (IND) is required for challenge studies in which a live organism is administered to subjects to study the pathogenesis of disease or the host response to the organism. While the challenge organism is not intended to have a therapeutic purpose, there is intent to affect the structure or function of the body. Consequently, the FDA considers the organism to be both a biological product and a drug and therefore subject to the corresponding regulatory requirements.^52^ As per the Federal Food, Drug, and Cosmetic Act, current good manufacturing practice (CGMP) must be in effect for the manufacture of investigational drug used during phase 1 clinical trials.^53^*C*. *parvum* human challenge studies (Table 2) were conducted in the US during 1990s and early 2000s with oocysts purified from experimentally infected neonatal calves. At the time, there was no requirement for an IND application for the challenge organism. Currently, there is no suitable robust manufacturing process available to produce *Cryptosporidium* oocysts ex vivo. A major barrier to producing large quantities of oocysts ex vivo has been the lack of a robust and reproducible in vitro culture system, although a hollow fiber continuous culture setup has been described with *C*. *parvum*.^54^ Our attempts to establish a robust hollow fiber *C*. *parvum* in vitro culture system were unsuccessful. The proposed alternate approach is by obtaining a purified *C*. *parvum* (Iowa isolate) oocysts from experimentally infected neonatal calves (a non-GLP source, Good Laboratory Practice). Oocysts are, then, surface sanitized, quality tested, and released by a GMP facility for use in the establishment of CHIM. We have also developed a surface sanitization protocol using peracetic acid to inactivate potential microbial and viral contaminants and demonstrated that this process is effective while having limited impact on *C*. *parvum* oocyst viability (Jumani et al., unpublished). *Cryptosporidium* oocysts can withstand peracetic acid treatment in contrast to other organisms.^55,56^ Peracetic acid is one of the most effective organic peroxide broad-spectrum biocide agents. It has been cleared by FDA as a sanitizer for direct food/food contact surfaces and recommended by CDC for the disinfection and sterilization of healthcare facilities and equipment, including reusable medical and dental devices. To confirm the effectiveness of the sanitization procedure, we have tested the oocysts treated with peracetic acid for the presence of microbial contaminants and shown that the sanitized oocysts do not contain any viable aerobic or anaerobic microorganisms as determined by regulatory guidelines. Furthermore, to confirm the effectiveness of the sanitization procedure on viral contaminants, purified oocysts were artificially contaminated with six different types of model viruses. The peracetic acid treatment reduced the infectivity of the spiked viruses to below the limit of detection. Currently, we are evaluating the logistics and feasibility of releasing *C*. *parvum* oocysts under GMP for CHIM studies.

### *C. parvum* Oocysts Gradually Lose Viability with Storage

Despite being highly resistant to harsh disinfection conditions, reliable cryopreservation of *Cryptosporidium* oocysts has been a long-standing challenge. A cryopreservation method for *C*. *parvum* oocysts has been recently developed,^57,58^ but the scalability and impact of cryopreservation to elicit human infection has not been evaluated. Currently, the routinely used storage condition for *C*. *parvum* oocysts is in aqueous suspension at 2–8 °C for 4–6 months. As the oocyst suspension ages, the viability reproducibly decreases and thus, the potential to induce an infection also decreases. Consequently, to ensure consistent infectivity in the clinic, CHIM will require a fresh batch of GMP oocysts every few months and may need to adjust the oocyst dose for loss of viability over time.

### Majority of Clinical Infections Are Caused by *C. hominis* and Anthroponotic *C. parvum* Strains

Epidemiologic studies have revealed that the majority of clinical infections in the endemic countries is caused by *C*. *hominis* (∼80%) and anthroponotic *C*. *parvum* (∼10%) isolates.^59−61^ The proposed high-dose oocyst human challenge model uses a *C*. *parvum* Iowa isolate, a zoonotic species which can cause a profuse watery diarrhea in both cattle and humans. Therefore, effectiveness of NCE in the CHIM may not directly reflect the efficacy against the most predominant clinical species. Though *C*. *parvum* and *C*. *hominis* share ∼96% nucleotide identity,^62,63^ it is critical to make sure the molecular target is conserved across *Cryptosporidium* species and determine the activity of NCE against *C*. *hominis* in early preclinical drug discovery stages. Several promising NCEs have been reported to have similar potency against *C*. *parvum* and *C*. *homins* in vitro suggesting the molecular target is conserved across these two species.^16,26,27,29^

### No Clear Relationship between the *C. parvum* Oocyst Infective Dose and Clinical Illness in Healthy Adults Has Been Established

The *C*. *parvum* Iowa isolate human challenge studies described in the literature have demonstrated that this isolate is capable of inducing infection in up to 100% of healthy volunteers with adequate doses of oocysts, but not all infected volunteers will develop diarrhea or other gastrointestinal (GI) symptoms (Table 2).^43,44,46−48^ In one study, 100% of healthy volunteers (*n* = 7) receiving ≥1000 *C*. *parvum* oocysts, that is, approximately 10 times above ID_50_ (infective dose) developed infection as measured by fecal oocyst shedding; 71% had enteric symptoms, but of these only 29% had diarrheal illness.^46^ The absence of a clear relationship between infective dose and diarrheal illness in healthy adults poses a challenge for using CHIM to demonstrate efficacy in improving diarrheal syndrome. It is likely that the positive health status of CHIM participants contributes to this variability. Multiple factors contribute to the susceptibility of the host to clinical manifestations such as host immune status, gut health, GI microbiota, the virulence of the *C*. *parvum* isolate and prior exposure to *Cryptosporidium*. Our proposed strategy is to use a high oocyst dose to increase the probability of infection and clinical symptoms in CHIM participants. We anticipate that ideally robust parasitological infection will be observed. However, to test efficacy of NCE, sufficient and consistent clinical illness along with parasitological infection in a significant proportion of healthy adults may be needed.

### Risk/Benefit Consideration for Participants

Aside from a long-term philanthropic contribution to the development of novel therapies for cryptosporidiosis, there is no direct benefit expected for healthy adults participating in a CHIM study. The risks to healthy participants may include GI cryptosporidiosis with mild to severe diarrhea, asymptomatic infections, persistent or recurrent illness, and possible secondary transmission. Extraintestinal manifestations in immunocompetent healthy adults have not been described in the published *Cryptosporidium* human challenge studies. Both symptomatic and asymptomatic infections may result in secondary transmission to household members and other contacts. Overall, the risks to participants and their contacts can be appropriately addressed in a clinical trial protocol for a CHIM study. In comparison, in longitudinal studies of adult outbreak-associated cryptosporidiosis, medium to long-term sequelae after resolution of the acute infection included diarrhea, abdominal pain, nausea, fatigue, headache, and joint pain.^64,65^ These long-term sequelae were more prevalent following infection with *C*. *hominis* than *C*. *parvum*.^66,67^ The impact of nitazoxanide treatment on long-term sequelae is unknown. In general, the interpretation of self-reported data from outbreak-associated cohorts requires caution given the potential for bias toward those most adversely affected and those who attributed postacute symptoms to acute cryptosporidiosis. Further, no such long-term sequelae have been described in the published *C*. *parvum* CHIM studies (Table 2). However, long-term follow up beyond 6–8 weeks was not conducted in most of these studies, but can be potentially monitored in future CHIM studies. Recently, an association between *Cryptosporidium* infections and GI cancers have been proposed, but a causal relationship has not been established.^68,69^ In a 2015, *C*. *muris* challenge study, two subjects with persistent oocyst shedding were successfully treated with nitazoxanide at 200 mg twice a day for 3 days, and the infection was resolved in both subjects, demonstrating the potential of nitazoxanide as a rescue drug.^51^ We propose to administer nitazoxanide to all participants in whom infection was elicited at the conclusion of the study or earlier in case of persistent or severe diarrhea to eliminate any remaining infection and decrease potential long-term risks.

### Uncertain Translatability of NCE Efficacy in a CHIM to Pediatric Cryptosporidiosis Diarrhea

In immunocompetent adults, *Cryptosporidium* infection causes self-limiting GI illness and symptoms most often completely self-resolve within 1–2 weeks (Table 2). In contrast, *Cryptosporidium* infection in young children, especially the malnourished or otherwise immunocompromised, is associated with life-threatening diarrhea with severe morbidity and mortality.^6−8^ In this vulnerable patient population, *Cryptosporidium* infection is often associated with persistent diarrhea (>14 days) leading to a significant adverse effect on linear (height) growth and nutritional shortfalls.^11,70^ Young children with cryptosporidiosis have more severe inflammation as measured by fecal lactoferrin levels as compared to adult volunteers.^48^ This may be due to various factors, including more severe diarrheal illness in children than healthy adults, presence of other enteric pathogens, nutritional status, gut health, sensitivity to fluid loss, and also differences in the virulence of *Cryptosporidium* isolates. Overall, cryptosporidiosis induced experimentally in healthy adults is not the same as the disease observed in the pediatric patients especially with respect to host health status, severity of diarrheal illness and complexity of pathogenesis. However, since the human challenge model recapitulates logarithmic parasite replication in the GI tract leading to fecal oocyst shedding, acute watery diarrhea, and other GI symptoms similar to pediatric patients, the CHIM is a scientifically robust and efficient approach to assess promising antiparasitic agents.

## Summary

*Cryptosporidium* is the second leading cause of diarrhea in young children and a major contributor for diarrheal deaths in LMICs. While cryptosporidiosis disproportionately affects young children, establishment of an adult CHIM is a scientifically robust and efficient way to assess novel antiparasitic agents with relatively less safety risk. Following a standard phase 1 with NCE in healthy adults, the *Cryptosporidium* CHIM would be a stepping stone to pediatric trials. It should help establishing a prospect of benefit for NCEs in healthy adults before advancing to the vulnerable pediatric population; derisking investment in juvenile toxicology studies; and providing PK/PD data to inform dose selection for pediatric trials.

Three key safety pillars of the proposed *C*. *parvum* CHIM studies protect participating healthy adults. First, a GMP-compliant oocyst manufacturing process to be established with sanitization, testing and batch release of oocysts as an investigational medical product. Second, the safety experience from several published CHIM studies.^43,44,46−48^ And finally, *C*. *parvum* infection in healthy adults causes a self-limiting illness and an effective rescue medication is available. A *Cryptosporidium* CHIM has the potential to accelerate the development of both new therapeutics and vaccines against cryptosporidiosis. The recent accomplishments in early drug discovery and availability of a *Cryptosporidium* controlled human infection model offer a compelling vision toward enabling a much-needed parasite-specific treatment for young children suffering from the debilitating effects of cryptosporidiosis.
